# MRI-based treatment plan simulation and adaptation for ion radiotherapy using a classification-based approach

**DOI:** 10.1186/1748-717X-8-51

**Published:** 2013-03-06

**Authors:** Christopher M Rank, Christoph Tremmel, Nora Hünemohr, Armin M Nagel, Oliver Jäkel, Steffen Greilich

**Affiliations:** 1 German Cancer Research Center (DKFZ), Division of Medical Physics in Radiation Oncology, Im Neuenheimer Feld 280, 69120 Heidelberg, Germany; 2 German Cancer Research Center (DKFZ), Division of Medical Physics in Radiology, Im Neuenheimer Feld 280, 69120 Heidelberg, Germany; 3 Heidelberg University Hospital, Department of Radiation Oncology, Im Neuenheimer Feld 400, 69120 Heidelberg, Germany

**Keywords:** Magnetic resonance imaging, Ion radiotherapy, Ion beam therapy, Treatment planning, Simulation, Plan adaptation, Pseudo CT, Classification, Ultrashort echo time

## Abstract

**Background:**

In order to benefit from the highly conformal irradiation of tumors in ion radiotherapy, sophisticated treatment planning and simulation are required. The purpose of this study was to investigate the potential of MRI for ion radiotherapy treatment plan simulation and adaptation using a classification-based approach.

**Methods:**

Firstly, a voxelwise tissue classification was applied to derive pseudo CT numbers from MR images using up to 8 contrasts. Appropriate MR sequences and parameters were evaluated in cross-validation studies of three phantoms. Secondly, ion radiotherapy treatment plans were optimized using both MRI-based pseudo CT and reference CT and recalculated on reference CT. Finally, a target shift was simulated and a treatment plan adapted to the shift was optimized on a pseudo CT and compared to reference CT optimizations without plan adaptation.

**Results:**

The derivation of pseudo CT values led to mean absolute errors in the range of 81 - 95 HU. Most significant deviations appeared at borders between air and different tissue classes and originated from partial volume effects. Simulations of ion radiotherapy treatment plans using pseudo CT for optimization revealed only small underdosages in distal regions of a target volume with deviations of the mean dose of PTV between 1.4 - 3.1% compared to reference CT optimizations. A plan adapted to the target volume shift and optimized on the pseudo CT exhibited a comparable target dose coverage as a non-adapted plan optimized on a reference CT.

**Conclusions:**

We were able to show that a MRI-based derivation of pseudo CT values using a purely statistical classification approach is feasible although no physical relationship exists. Large errors appeared at compact bone classes and came from an imperfect distinction of bones and other tissue types in MRI. In simulations of treatment plans, it was demonstrated that these deviations are comparable to uncertainties of a target volume shift of 2 mm in two directions indicating that especially applications for adaptive ion radiotherapy are interesting.

## Background

Ion radiotherapy offers the opportunity of highly conformal irradiation of tumors applying high doses to the tumor volume while sparing the surrounding tissue and organs at risk [1]. In order to achieve this benefit, sophisticated treatment planning and simulation are required. Major uncertainties in the ion radiotherapy treatment process are interfractional and intrafractional changes in patient anatomy, for instance organ and tumor movement, tumor shrinkage, filling of air cavities and loss of weight. Today, the gold standard for radiotherapy treatment plan simulation is Computed Tomography (CT). A stoichiometric Hounsfield look-up table (HLUT) is used to derive water equivalent path length (WEPL) values for ions from the measured X-ray CT numbers.

Here we investigate the potential of Magnetic Resonance Imaging (MRI) as an alternative to CT-based treatment planning and simulation alone, especially for applications in adaptive ion radiotherapy. In contrast to photons, depth-dose distributions of ions are far more sensitive to whether the tissue class has been assigned correctly. This study presents therefore a reliable test if MRI-based treatment plan simulation is generally feasible.

As MRI is based on the effect of nuclear magnetic resonance, patients are not exposed to ionizing radiation during measurements. Therefore MRI provides the opportunity of patient examinations before each fraction for validation and adaptation of treatment plans. In the future, even real-time MRI-guided ion radiotherapy is imaginable, analogue to current studies with X-rays using a hybrid MRI linac modality [2,3].

The major challenge in the application of MRI for treatment plan simulation is the fact that there exists no physical relationship between MR signal intensities and WEPL values. The MR signal depends mostly on the density of water and fat protons as well as tissue relaxation characteristics, whereas WEPL values are correlated to the electron density and the mean excitation energy. A two-step approach for the MRI-WEPL conversion is proposed here. First so-called pseudo CT (p CT) numbers are derived from MR signal intensities followed by a conversion to WEPL values using an empirical HLUT.

In the literature two different approaches for the derivation of p CT numbers from MR images can be found that are suggested for conventional photon radiotherapy treatment plan simulation [4-12] and MR/PET attenuation correction [13-17]: a first approach deals with atlas-based methods that employ non-rigid registration between a template MR image and the patient’s MR image to warp a template CT image to the patient’s anatomy. A second approach consists of segmentation-based methods that use a voxelwise classification of tissue into several classes that are correlated with certain bulk CT numbers.

**Figure 1:**
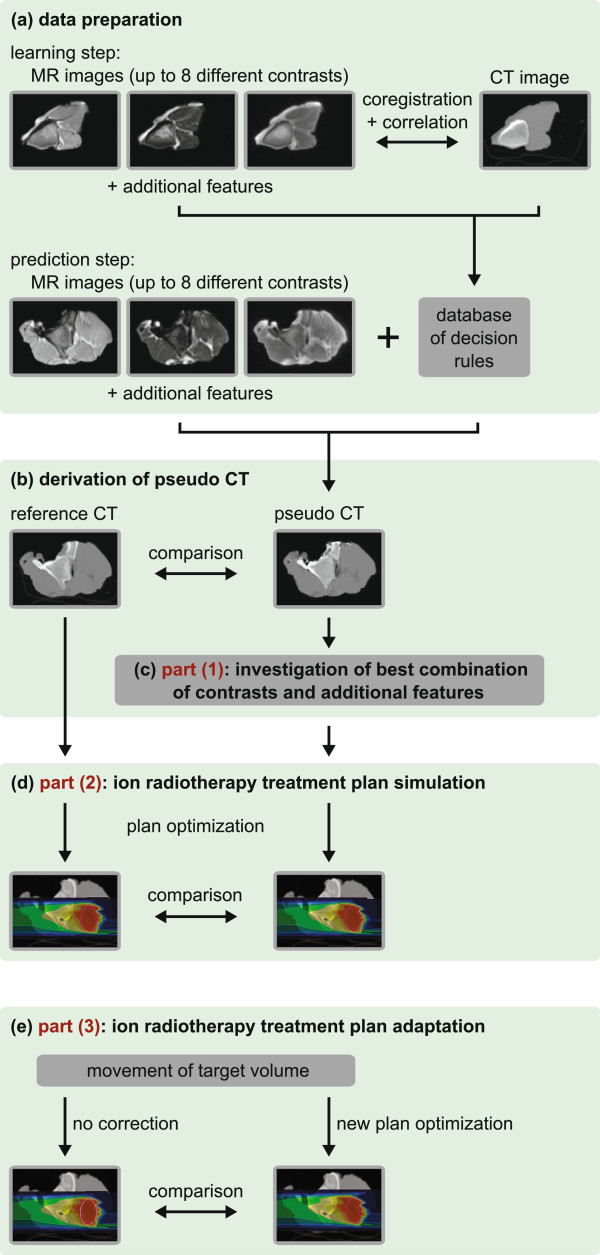
**Experiments and workflow of MRI-based ion radiotherapy treatment plan simulation and adaptation.(a)** data preparation for classification; **(b)** derivation of p CT numbers from MR images; **(c)** cross-validation study for investigating the best combination of contrasts and additional features; **(d)** ion radiotherapy treatment plan simulation; **(e)** ion radiotherapy treatment plan adaptation.

In this work the potential of the second approach is investigated using a classifier that is based on discriminant analysis. After data preparation (Figure 1(a)), each voxel in a MR image can be associated with a certain p CT number (Figure 1(b)). The classification method was chosen as it allows a patient-specific voxelwise prediction of p CT numbers. In comparison to atlas-based methods, it might perform better handling anatomical changes that cannot be described by a translation or deformation of voxels, e. g. the filling of an air cavity with mucus. However, it is not the aim of this study to compare both methods.

The study consists of three parts. (1) Appropriate MR sequences and parameters are evaluated by cross-validation studies of three tissue phantoms in order to minimize errors of p CTs compared to a reference CT (r CT) (Figure 1(c)). (2) The p CTs with lowest errors are used for ion radiotherapy treatment plan simulations (Figure 1(d)). For each phantom ion radiotherapy treatment plans are optimized to a planning target volume (PTV) on both p CT and r CT images. The resulting fields are then both calculated on r CT images and compared afterwards. (3) A target movement is simulated by shifting the irradiated PTV (Figure 1(e)). Ion radiotherapy plans adapted to the new position of the PTV are optimized on a p CT. The results are compared to optimizations on a r CT without taking account of the target shift.

## Material and methods

### Multimodal phantoms

As specific MR sequences were employed for the present study, which are not applied at clinical radiotherapy examinations, a retrospective evaluation of patient data was not possible. Therefore three pieces of pork meat were used as multimodal phantoms. These samples were composed of fatty tissue, muscle and bones to cover a large range of MR relaxation parameters and attenuation properties for photons and ions in living tissue (Table 1).

**Table 1:** MR- and CT-related parameters for tissue from literature

**Tissue**	**Relative electron**	**T1 [ms]**	**T2 [ms]**
	**density *ρ***_**e**_		
subcutaneous fat	0.951^*a*^	371^*d*^	133^*d*^
skeleton - yellow marrow	0.982^*a*^	365^*d*^	133^*d*^
brain - white matter	1.035^*a*^	1084^*c*^	69^*c*^
brain - gray matter	1.035^*a*^	1820^*c*^	99^*c*^
muscle	1.040^*a*^	1412^*c*^ - 1420^*d*^	32^*d*^ - 50^*c*^
kidney	1.041^*a*^	1194^*c*^	56^*c*^
blood	1.050^*a*^	1932^*c*^	275^*c*^
liver	1.050^*a*^	812^*c*^	42^*c*^
skeleton - cartilage	1.083^*a*^	1156^*c*^ - 1240^*d*^	27^*c*^ - 43^*c*^
skeleton - cortical bone	1.781^*a*^	140^*b*^ - 260^*b*^	0.42^*b*^ - 0.50^*b*^

^*a*^[18], ^*b*^[19], ^*c*^[20], ^*d*^[21]. All measurements were performed in vivo at 3T, values for cortical bone were measured at 1.5T and represent the relaxation time T2 ^*^.

### CT image acquisition

CT image acquisition was performed on a Siemens Somatom Definition Flash CT scanner. The X-ray spectra voltage was 140 kVp with additional tin filtration and an effective tube current-time product of 81 mAs. Images were reconstructed from raw data with a D30s kernel. The slice thickness was 1.0 mm and a field of view of 256 mm was chosen resulting in an in-plane resolution of 0.5 mm.

### MR image acquisition

MR images were acquired on a Siemens Magnetom Trio Tim 3T MRI scanner. For all samples a set of eight image series with various contrasts was created. The imaging of bones using standard MR sequences leads to very low signal intensities similar to air due to the very short T2 relaxation constant of bony tissue. Therefore a 3D ultrashort echotime (UTE) sequence with radial k-space sampling was employed, that allows for distinguishing bone tissue from air [19,22-24]. In addition, a 2D turbo spin echo (TSE) sequence with proton density- and T2-weighting and a 3D ultrafast gradient echo (MPRAGE) sequence with magnetization preparation for T1-weighting were used (Table 2). All sequences had the same field of view of 256 mm. The TSE and MPRAGE sequences had a nominal resolution of 1.0 × 1.0 × 2.0 mm^3^, whereas the nominal resolution of the UTE sequence was isotropic with 1.0 × 1.0 × 1.0 mm^3^. An automatic distortion correction filter and a correction of inhomogeneous coil illumination was activated in each sequence protocol.

**Table 2:** Parameter settings of MR sequences

**Sequence**	**Echo time**	**Repetition time**	**Flip angle**	**Scan time**	**Additional parameters**
	**[ms]**	**[ms]**	**[°]**	**[min]**	
TSE1	8.3	7500	90	4.8	
TSE2	8.3	7500	90	4.8	with fat saturation
TSE3	75.0	7500	90	3.8	
MPRAGE	2.38	2000	12	6.4	inversion time = 700 ms
UTE1	0.05	7.25	12	6.1	radial views = 50001
	4.91				
UTE2	0.05	7.25	60	6.1	radial views = 50001
	4.91				

### Image registration and masking

All acquired MR images were coregistered to their corresponding CT images with a rigid registration algorithm based on maximization of mutual information [25]. MR images were resampled to CT resolution in all three dimensions by linear interpolation to make a voxelwise correlation of images possible. Afterwards, voxels at the image edges consisting of air were removed by a threshold mask in order to reduce computing time for additional feature extraction and classification. The following calculations refer only to voxels within that mask.

### Additional feature extraction

Additional features were extracted for each voxel from its 26 surrounding voxels of a 3 × 3 × 3 box according to [10] and its location in order to augment the information provided by the signal intensities of voxels: 

• *box.mean*: the mean intensity of the surrounding box including the central voxel

• *box.sd*: the standard deviation of the surrounding box including the central voxel multiplied by the intensity of the voxel

• *dist.xyz*: the three absolute distances of the voxel to the center of the 3D imaging volume in each spatial dimension

• *dist.center*: the absolute distance of the voxel to the center of the 2D slice

Neighborhood-related parameters were employed for improving results at transitions, e. g. standard deviations of boxes at transitions were larger than at homogeneous sites. Extracting coodinate-related features assumed a certain symmetric spatial distribution of tissue classes, in particular a cylindrical symmetry for *dist.center* and a spherical symmetry for *dist.xyz*. Before calculating distances of voxels to the image center, coordinates of voxels were translated so that samples were centered in the coordinate system of images.

### Discriminant analysis

The idea of discriminant analysis is to assign one of *k* predefined classes *C*_1_,*C*_2_,…,*C*_*k*_ to an observation variables vector $X={\left(x_{1},x_{2},\ldots ,x_{p}\right)}^{\text{T}}\in {\mathbb{R}}^{p}$ with unknown class membership [26]. In the learning step a learning data set that consists of *n* observation variables vectors **X**_*i*_ labeled with their corresponding classes *C*_*i*_ is employed to derive a decision rule that can associate a new observation variables vector **X** with one of the *k* classes (Figure 1(a)). Class memberships of new observation variable vectors can then be predicted using the decision rule (Figure 1(b)).

Here, each observation variables vector **X**_*j*_ was composed of intensities of several MRI contrasts and additionally extracted features of one particular voxel *j* of the coregistered MR images. Class membership of a voxel was obtained from its CT number. Therefore the CT scale was divided into 128 sections each having a range of 32 HU and each voxel *j* was assigned with its appropriate CT class *C*_*j*_.

### Classification algorithm

For classification of voxels into CT classes the high dimensional discriminant analysis (HDDA) algorithm from the package “HDclassif” (version 1.2.1 [26,27]) implemented in R (The R Foundation for Statistical Computing, version 2.14.2 [28]) was chosen as a reliable, well-tested and efficient method. It is closely related to the well-known quadratic discriminant analysis (QDA). According to the classical Gaussian mixture model framework, HDDA assumes that class conditional densities are Gaussian. Thus, a certain variation of absolute MR signal intensities as observation variables, e. g. due to inhomogeneous coil illumination, was taken into account. To increase efficiency in computing time compared to QDA, the assumption is made that high-dimensional data live around subspaces with a lower dimension than *p*. Therefore the number of dimensions is reduced during the learning step and for each of the *k* classes only the dimensions which contain most information for discrimination from other classes are maintained. In the HDDA parameter settings the most general model *a*_*kj*_*b*_*k*_*Q*_*k*_*d*_*k*_ was used with a threshold for dimension selection of 0.2. In addition the datasets were scaled so that each observation variable was equally weighted having a mean value of 0 and a standard deviation of 1. The p CT number of a voxel was then obtained by the dot product of the posterior probabilities of a voxel for each of the *k* classes and the mean CT number of each class calculated from the learning dataset.

### Ion radiotherapy treatment plan simulation

**Figure 2:**
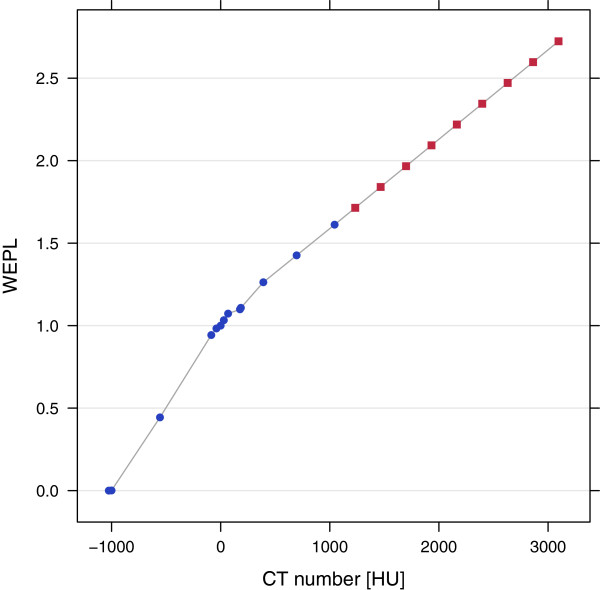
**Empirical Hounsfield lookup table for CT to WEPL conversion.** Blue dots represent measured values of Gammex tissue equivalent materials and red squares were extrapolated.

Ion radiotherapy plans based on r CT or p CT images were created using the treatment planning system VIRTUOS developed at DKFZ [29]. Prescribed physical doses were 3 Gy for protons and 1 Gy for carbon ions. Irradiation fields were optimized and simulated with the software TRiP98 from GSI, Darmstadt (Germany) [30,31]. A raster-scanning pencil beam for protons and carbon ions with a FWHM of 5 mm passing a 3 mm ripple filter was assumed. WEPL values were obtained from r CT or p CT images with an empirical HLUT that was derived from 140 kVp CT measurements of Gammex tissue equivalent materials (Figure 2).

### Ion radiotherapy treatment plan adaptation

In order to investigate the potential of MRI for treatment plan adaptation, i. e. to align plans with interfractional anatomical changes, a target volume movement was simulated by shifting the irradiated PTV of meat sample 2. Then, optimizations of carbon ion and proton treatment plans created in VIRTUOS and adapted to the target shift were performed on p CT images with TRiP98 and compared to a r CT optimization without plan adaptation.

## Results

### Derivation of pseudo CT numbers from MR images

For all three samples, CT images and MR images with eight different contrasts were acquired and coregistered. In a cross-validation study, for each sample a p CT was predicted from MR images using the other two samples for creating the learning database. 738 different combinations of contrasts and additional features as input of the observation variables vector **X** were tested for datasets with a reduced number of 32 images per sample. For all these combinations, the mean absolute errors MAE for masked voxels were calculated by a voxelwise comparison of p CT and r CT [10]:

(1) $$\text{MAE}=\frac{1}{n}\overset{n}{\underset{i=1}{\sum }}|{\text{p CT}}_{i}-{\text{r CT}}_{i}|.$$

**Figure 3:**
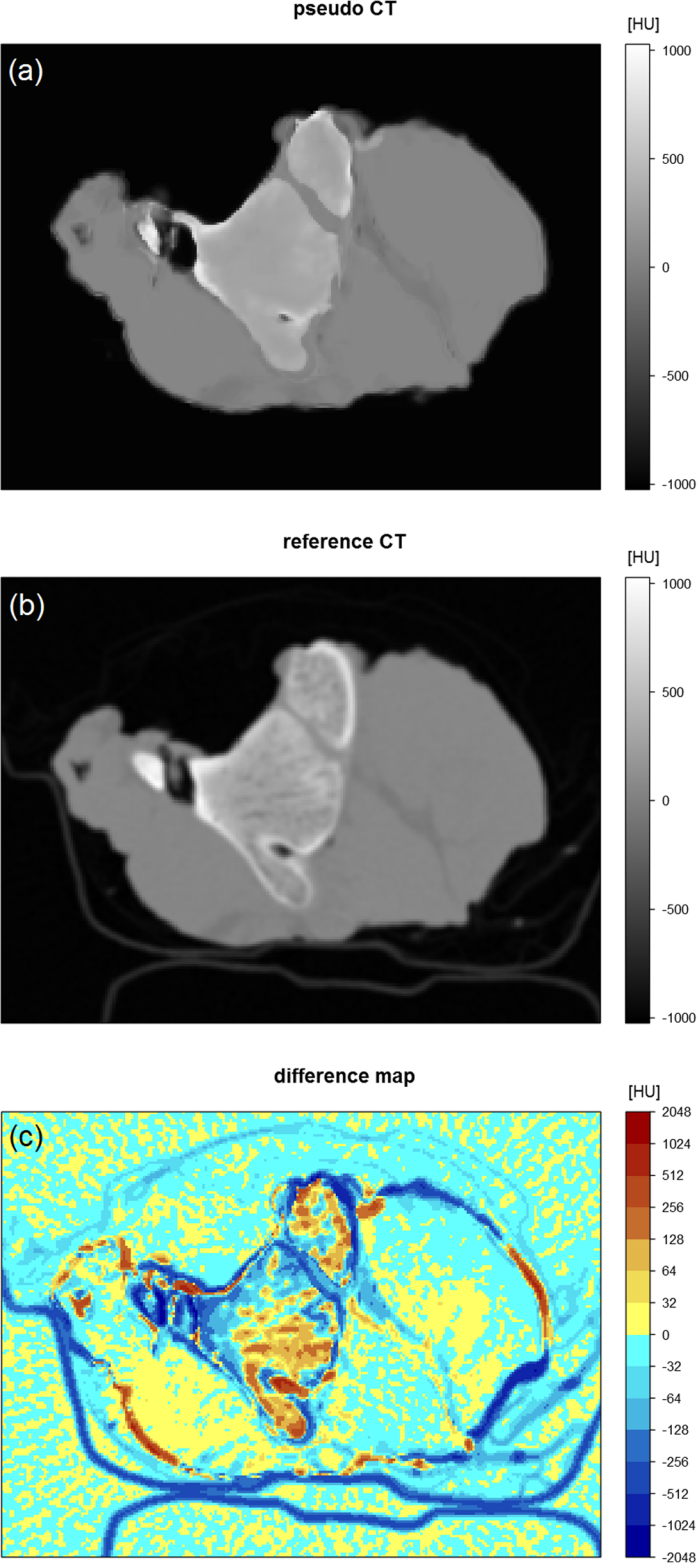
**Pseudo CT, reference CT and difference map of one slice of sample 2.(a)** pseudo CT using the best combination of contrasts and additional features. The sample was placed in a bowl made out of paper that gave no signal in MRI resulting in a wrong classification of these voxels as air; **(b)** reference CT; **(c)** difference map from data of **(a)** and **(b)**. In the logarithmic color scale, red pixels represent an overestimation and blue pixels an underestimation of the reference CT number.

The best results were achieved with a combination of the proton density weighted TSE sequence (TSE1) and the UTE 2 sequence each with additional features *box.sd* as well as *dist.center* resulting in a 7 dimensional observation variables vector **X** (Table 3). The mean absolute errors for such a combination for the full datasets were MAE_1_ = 81.0 HU, MAE_2_ = 95.2 HU and MAE_3_ = 90.1 HU for samples 1-3, respectively. Variations of absolute errors between voxels were large leading to standard deviations of absolute errors between 130 HU and 152 HU. The above described combination needed around 5 - 6 min of computing time for both the learning step and the prediction step (running on an Intel Core i5 processor with 4 cores for sets of 200 images for learning and 100 images for prediction). Each image was represented by a matrix of 43560 voxels. For further analysis, the r CT (Figure 3(b)) was subtracted from the p CT for each sample using the best combination of contrasts (TSE1, UTE2) and additional features (*box.sd*, *dist.center*) (Figure 3(a)) yielding a difference map (Figure 3(c)).

**Table 3:** Results of cross-validation study

**Contrasts**	**Additional features**	**Mean absolute**
		**error [HU]**
TSE1, UTE2	*box.sd*, *dist.center*	92.5
TSE1, UTE2	*box.sd*, *dist.xyz*	93.5
TSE1, UTE1	*box.sd*, *dist.center*	94.1
TSE1, UTE2	*box.sd*, *dist.center*, *dist.xyz*	95.8
TSE1, UTE2	*box.mean*, *box.sd*, *dist.center*	96.5
TSE1, UTE2	*box.mean*, *box.sd*, *dist.xyz*	96.5
TSE1, MPRAGE, UTE2	*box.sd*, *dist.center*	96.7
TSE1, TSE2, UTE2	*box.sd*, *dist.center*	97.0
TSE1, UTE1, UTE2	none	111.7
UTE1	*box.mean*, *box.sd*, *dist.xyz*	100.7
TSE1, MPRAGE	*box.mean*, *box.sd*, *dist.center*	115.7

Mean absolute errors of masked voxels for the 8 best combinations of contrasts and additional features out of 738 combinations in comparison to the best result without any additional feature as well as without TSE1 and UTE sequences. Results were obtained for reduced datasets of 32 images per sample.

In addition the mean errors of p CT per class ME_*cl*_ for all three samples were calculated:

(2) $${\text{ME}}_{\text{cl}}=\frac{1}{n_{\text{cl}}}\overset{n_{\text{cl}}}{\underset{i=1}{\sum }}{\text{p CT}}_{i,\text{cl}}-{\text{r CT}}_{i,\text{cl}}.$$

**Figure 4:**
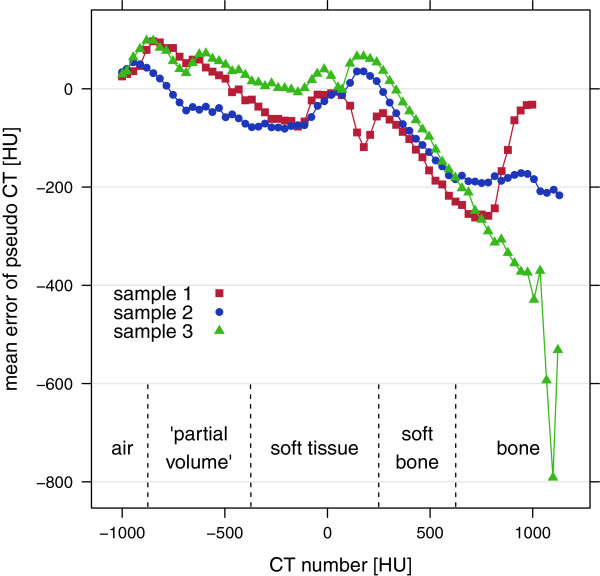
**Mean errors of p CT per class for the three samples.** The best combination of contrasts and additional features was used for p CT prediction.

The dependence of MEs on CT numbers was similar for all three samples and showed a strong systematical underestimation for bony tissue (Figure 4).

### Ion radiotherapy treatment plan simulation

Pseudo CT numbers and corresponding errors of the three samples were translated into pseudo WEPL values and errors by means of the 140 kVp empirical HLUT (Figure 2). From this an estimate of the mean deviation of water equivalent thickness (WET) for five tissue classes, namely air, “partial volume”, soft tissue, soft bone and bone was calculated:

(3) $$\Delta {\text{WET}}_{\text{tissue}}=\Delta {\text{WEPL}}_{\text{tissue}}\times d,$$

with *d* as tissue thickness. For evaluation of variations of *Δ*WET the standard error per tissue for a beam passing three different thicknesses was computed assuming an irradiation in a lateral direction where the image resolution was 0.5 mm. Similar to the calculation of mean errors of p CT per class, very dense bone classes were significantly underestimated, whereas the air and the “partial volume” class exhibited an overestimation. Soft tissue was in good agreement with reference values (Figure 4 and Table 4).

**Table 4:** Mean errors of p CT and estimated mean deviations of WET for different tissue types

**Tissue**	**Mean error of**	***Δ*WET**_**1*cm***_	***Δ*WET**_**5cm**_	***Δ*WET**_**10cm**_
	**p CT [HU]**	**[mm]**	**[mm]**	**[mm]**
air	43.3	0.4 ± 0.3	1.8 ± 0.6	3.6 ± 0.9
“partial volume”	28.6	0.3 ± 0.7	1.4 ± 1.5	2.9 ± 2.1
soft tissue	-1.4	-0.02 ± 0.3	-0.1 ± 0.6	-0.2 ± 0.9
soft bone	-63.2	-0.4 ± 0.3	-1.8 ± 0.6	-3.7 ± 0.9
bone	-223.1	-1.2 ± 0.4	-6.0 ± 0.8	-11.9 ± 1.1

The best combination of contrasts and additional features was used for calculations.

**Figure 5:**
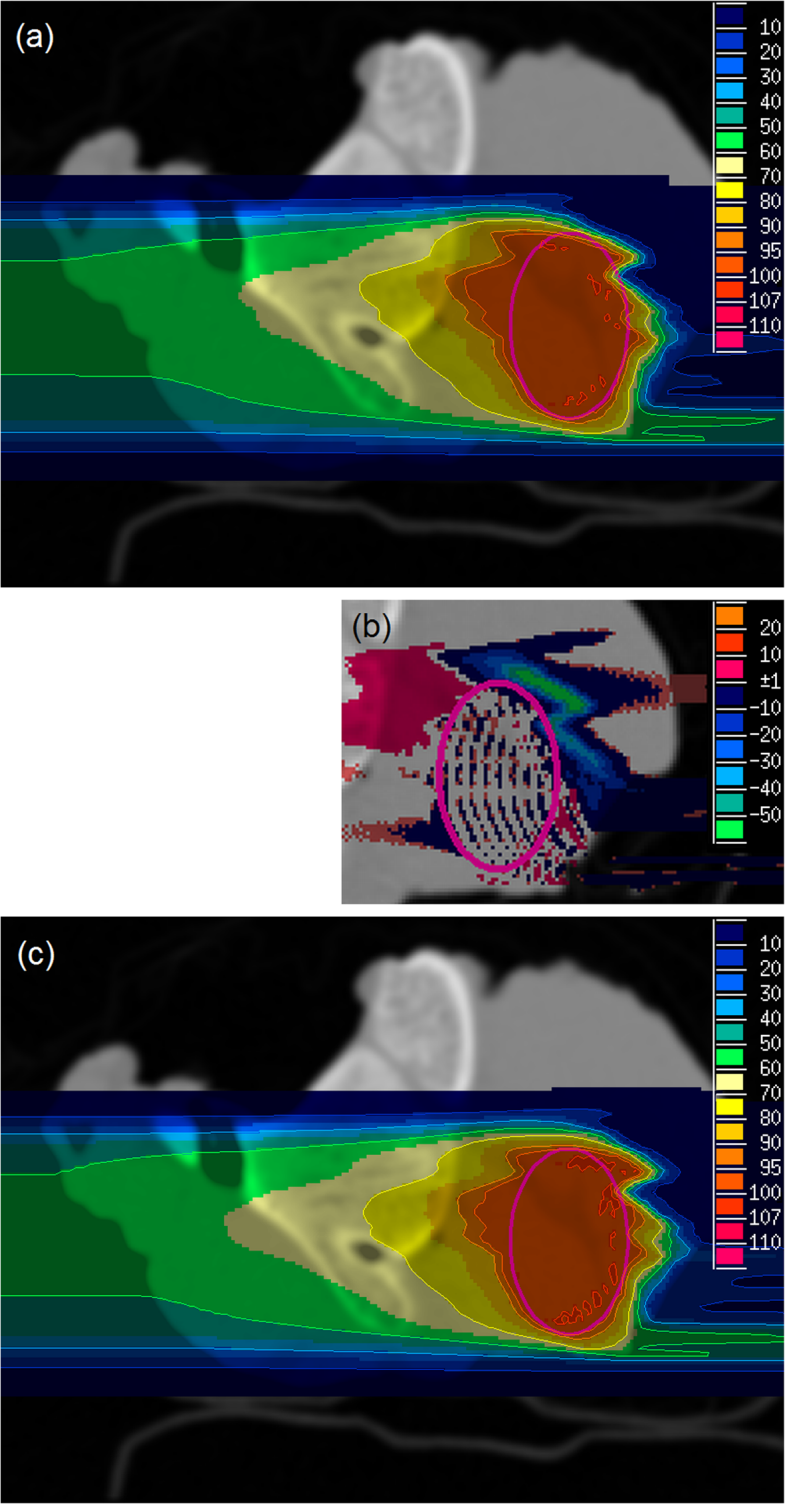
**Carbon ion plans of sample 2.(a)** optimized on p CT and recalculated on r CT; **(b)** dose difference of **(a)** and **(c)**; **(c)** optimized and calculated on r CT. The PTV size was 17 × 26 × 16mm^3^ and the unit of color scale is percent of the prescribed physical dose.

For treatment plan simulations, PTVs were placed in each sample at a position considered to be the most challenging, i. e. the ion beam had to pass bony structures. After p CT and r CT conversion to WEPL values, proton and carbon field optimizations were run on both MRI-based p CT images as well as on r CT images for all three samples. The fields optimized on MRI-based p CTs were recalculated on r CTs that were considered to be the gold standard (Figure 5(a)). The results were compared with plans optimized and calculated on r CT images (Figure 5(b), (c)).

**Figure 6:**
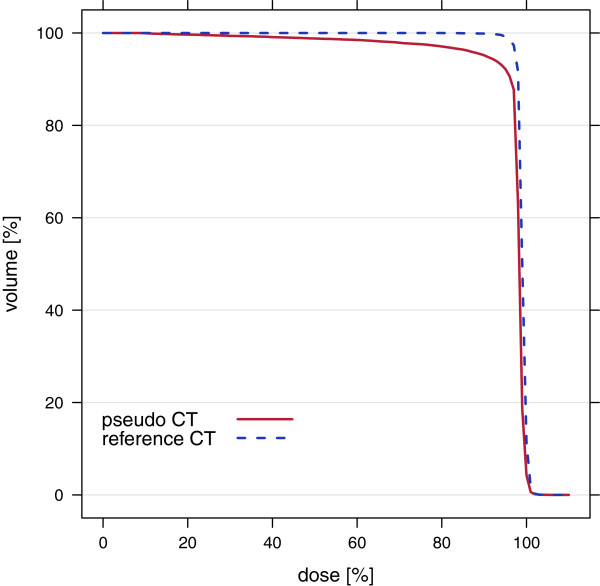
**Dose-volume histograms of sample 2 for carbon plans.** Optimized on MRI-based p CT and recalculated on r CT (red curve) as well as optimized and calculated on r CT (blue curve).

Dose-Volume histograms (DVH) were computed for the fields optimized on MRI-based p CTs and recalculated on r CTs (Figure 6 red curve) as well as for fields optimized and calculated on r CTs (Figure 6 blue curve). Table 5 shows the corresponding dose statistics parameters mean dose of PTV and volume of PTV with dose less than 90% of prescribed dose.

**Table 5:** Dose statistics parameters of carbon ion and proton treatment plans

		**p CT optimization**		**r CT optimization**	
**Sample type**	**Beam modality**	**Mean**	**Volume with dose**	**Mean**	**Volume with dose**
		**dose [%]**	**<90 *% *[ *% *]**	**dose [%]**	**<90 *% *[ *% *]**
meat sample 1	carbon	96.5	8.4	99.0	0.7
	proton	96.2	8.3	99.3	0.0
meat sample 2	carbon	96.7	4.6	99.0	0.1
	proton	96.2	6.0	98.9	0.4
meat sample 3	carbon	97.4	3.4	98.8	0.3
	proton	97.3	2.4	98.8	0.2

Plans were optimized on pCT as well as rCT and both optimizations were recalculated on r CT for comparison of parameters.

### Ion radiotherapy treatment plan adaptation

**Figure 7:**
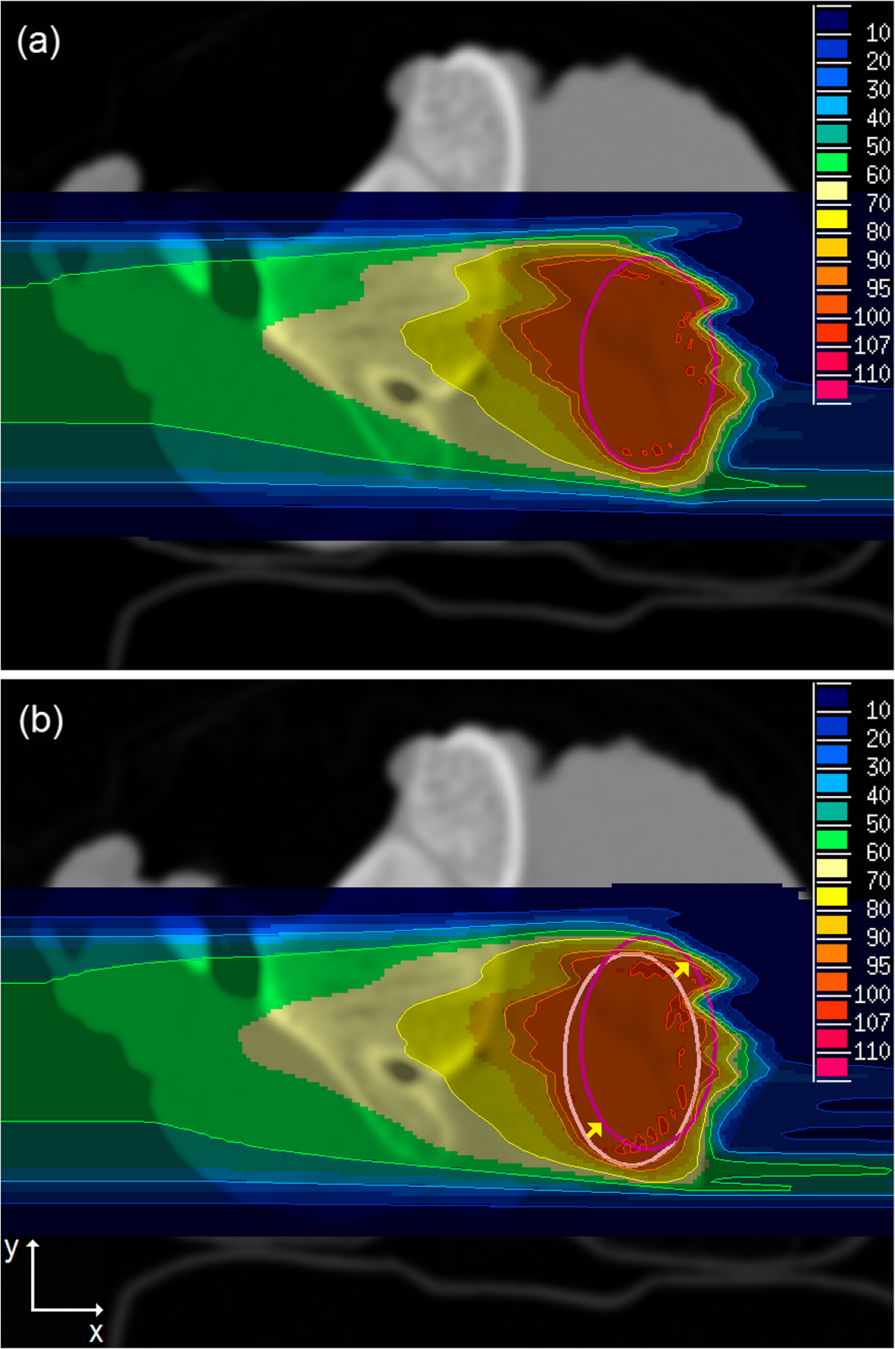
**Carbon ion plans of sample 2 with shifted PTV.** The old PTV (light magenta) was shifted 2.0 mm in *x*- and 2.0 mm in *y*-direction to a new position (dark magenta): **(a)** adapted plan optimized on p CT after PTV shift and recalculated on r CT, **(b)** optimized on r CT before PTV shift and recalculated on r CT after PTV shift without plan adaptation. The unit of color scale is percent of the prescribed physical dose.

The irradiated PTV of sample 2 was shifted 2.0 mm in *x*- and 2.0 mm in *y*-direction for simulating a target volume movement (Figure 7(b)).

**Figure 8:**
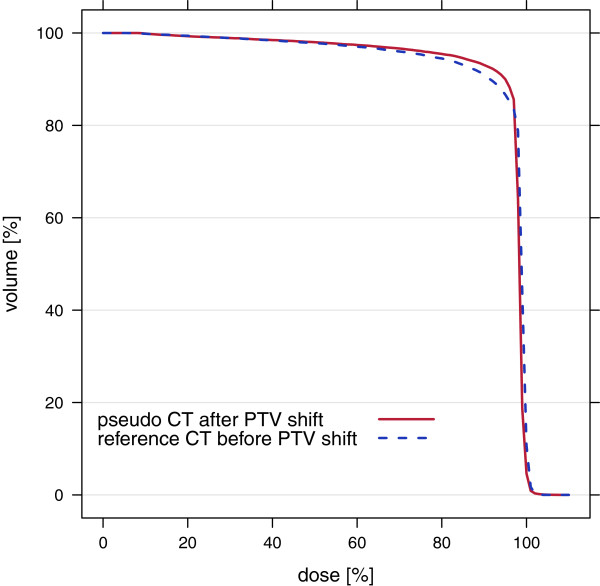
**Dose-volume histograms of sample 2 for carbon plans with shifted PTV.** Optimized on p CT after PTV shift (red curve) and on r CT before PTV shift without plan adaptation (blue curve). Both plans were recalculated on the r CT after PTV shift.

Proton and carbon field optimizations were run on the p CT using a plan adapted to the shifted PTV. The optimized fields were recalculated on the r CT with the shifted PTV (Figure 7(a)). The results were compared to fields optimized on r CT before PTV shift and recalculated on the r CT after PTV shift without plan adaptation (Figure 7(b)). DVHs were computed for both optimizations (Figure 8). Adapted p CT optimizations showed slightly better results than non-adapted r CT optimizations having comparable mean doses of PTV and 2.0 - 2.4% smaller volumes of PTV with doses less than 90% of prescribed dose.

## Discussion

### Derivation of pseudo CT numbers from MR images

The cross-validation study with different combinations of contrasts and additional features revealed remarkable results: (1) the UTE sequence was essential for the distinction of bony tissue and air and could reduce MAE by 20% (Table 3). (2) Proton density weighted images seemed to contain the most information for the derivation of p CT values compared to all other tested contrasts besides the UTE sequence. (3) Additional feature extraction could reduce the MAE by 17%. In general, p CT numbers of water-like soft tissue around 0 HU had the lowest absolute errors compared to the r CT (Figure 4). However, MEs could not be smaller than ± 16 HU in average as the CT scale was divided into sections of 32 HU.

Compact bone classes showed a strong systematical underestimation. One reason for this comes from an imperfect signal discrimination of bones and other tissue types using the UTE sequence. In particular fatty-tissue showed a strong signal at UTE images that were acquired at very short echo times. Another cause for the underestimation is related to clipping errors. Bone tissue classes represented the classes with highest CT numbers. Therefore either a correct classification or an underestimation was possible producing too low CT numbers on average.

Largest errors could be found at transitions from air to soft tissue, soft tissue to bone and air to bone. These intermediate regions between different tissue classes included single voxels with deviations of more than 1500 HU. In particular partial volume effects, errors of registration and image resampling by linear interpolation were responsible for these large errors leading to a wrong classification on the CT scale.

In the extraction of additional features, the *box.sd* feature contributed most to the improvements (Table 3). Near borders the standard deviation of a box surrounding a voxel was higher than in homogeneous tissue. This additional information used as a further dimension of the observation variables vector improved discrimination of “partial volume” and other tissue classes and spots at borders with deviations larger than 500 HU could be reduced significantly. The coordinate-related features *dist.xyz* and *dist.center* both also decreased the MAE, showing that the spatial distribution of several tissue classes had certain degrees of cylindrical and spherical symmetries.

In the following, several opportunities are proposed for improving the classification results: 

• an extended set of samples (more than two) should be employed for creating the learning database of decision rules to compensate for anomalies and differences between samples

• more suitable MRI contrast parameters may have to be found for a further improved discrimination of tissue classes

• an UTE sequence with fat saturation might improve the distinction of bones and other tissue, in particular fatty tissue

• a better correction of inhomogeneous coil illumination than the implemented filter might decrease the variances of MR signal intensities of the different classes

• further additional features, e. g. larger box sizes of 7 x 7 x 7 voxels for mean and standard deviation might reduce the effects of partial volume and interpolation leading to smaller errors at tissue transitions

• CT scale may be divided into non-equidistant sections or other ways of class determination may exist, e. g. dependent on spatial location of a voxel combined with its CT number

• more sophisticated discrimination tools like non-parametric discriminant analysis or support vector machines may have to be used instead of HDDA

### Ion radiotherapy treatment plan simulation

In the simulation of ion radiotherapy treatment plans, MRI-based p CT optimized plans had small deviations compared to r CT optimized plans that were assumed to be the gold standard. Dose coverage of PTVs was slightly better for r CT optimizations than for p CT optimizations (Figure 5 and Table 5). In particular, mean doses of PTVs were 1.4 - 3.1% higher, whereas volumes of PTVs with doses less than 90% of prescribed dose were 2.2 - 8.3% smaller. Especially distal regions of PTVs showed an underdosage in p CT optimizations. This was expected as the ions had to pass bony structures that were systematically underestimated in p CT images.

The DVHs also revealed a better dose coverage of PTVs for r CT optimizations compared to p CT optimizations (Figure 6). These results demonstrated that the classification method for deriving p CT numbers from MR images performed well in the soft tissue region, whereas for dense materials like compact bones errors were large. However, the effect of these large errors on dose dostributions of treatment plan simulations using p CT for optimization was relatively small. This can be explained by the composition of bones having only a thin outer layer of compact bone. Therefore the proportion of voxels with these large errors was small.

### Ion radiotherapy treatment plan adaptation

MRI-based treatment plan adaptation was tested by simulating a movement of target volume. A plan optimized on p CT and adapted to the PTV shift was compared to a plan optimized on r CT before PTV shift. The adapted plan optimized on p CT showed a slightly better dose coverage of the shifted target volume as the r CT plan without plan adaptation (Figures 7, 8).

This result indicated that the error introduced by using a p CT for optimization was comparable to the error resulting from a target volume shift of 2.0 mm in *x*- and *y*-direction. In this particular case, MRI examinations before each fraction and a treatment plan adaptation to interfractional changes of anatomy or tumor movements would improve dose coverage of PTV for target volume shifts larger than 2.0 mm in two directions. In order to reduce errors using p CT, a combination of the original r CT with a p CT might be employed for adaptation of treatment plans. As the bone tissue class had largest deviations in p CT images and anatomical changes in bony structures are uncommon, solely soft tissue regions of the r CT in which anatomical changes are found in MRI could be substituted by p CT values. However, this approach might also introduce registration errors and a careful evaluation of advantages and disadvantages is necessary.

## Conclusions

In this study the potential of MRI for treatment plan simulation and adaptation in ion radiotherapy was investigated. It was shown that a MRI-based derivation of p CT values using a statistical classification approach is feasible although no physical relationship between MR signal intensities and X-ray CT numbers exists. Results obtained in cross-validation studies of three tissue samples showed a strong underestimation of compact bone classes resulting from an imperfect distinction of bones and other tissue types applying the UTE MR sequence. In simulations of treatment plans these deviations revealed mean doses of PTVs being 1.4 - 3.1% smaller for p CT optimizations compared to r CT. Considering adaptation of treatment plans, these deviations corresponded to uncertainties introduced by an interfractional target volume shift of 2 mm in two directions.

In the future MRI may complement the treatment planning process for ion radiotherapy and improve the accuracy so as to reap the rewards of highly conformal irradiation of tumors with charged particles. Especially applications for adaptive ion radiotherapy seem to be interesting as MRI examinations before each fraction would give the opportunity of adaptation to interfractional changes of anatomy without additional dose to the patient.

## Competing interests

The authors declare that they have no competing interests.

## Authors’ contributions

CMR designed the study, carried out the MRI and CT measurements, developed the framework for data evaluation, performed the statistical analysis and drafted the manuscript. CT measured data for the HLUT for CT to WEPL conversion. NH supported the CT measurements and helped to draft the manuscript. AMN supported the MRI measurements and helped to draft the manuscript. OJ participated in the design of the study and helped to draft the manuscript. SG initiated the study, participated in its design and coordination and helped to draft the manuscript. All authors read and approved the final manuscript.
